# Ultra-Responsive MEMS Sensing Chip for Differential Thermal Analysis (DTA)

**DOI:** 10.3390/s23031362

**Published:** 2023-01-26

**Authors:** Haozhi Zhang, Hao Jia, Weiwen Feng, Zao Ni, Pengcheng Xu, Xinxin Li

**Affiliations:** 1 State Key Laboratory of Transducer Technology, Shanghai Institute of Microsystem and Information Technology, Chinese Academy of Sciences, Shanghai 200050, China; 2 School of Microelectronics, University of Chinese Academy of Sciences, Beijing 100049, China

**Keywords:** MEMS thermopile, differential thermal analysis, DTA, single-crystal silicon thermocouples, MIS process

## Abstract

Ultra-responsive single-crystal silicon MEMS thermopiles for differential thermal analysis (DTA) are developed. Facilitated by a unique “microholes interetch and sealing (MIS)” technique, pairs of suspended thermopiles are batch fabricated in a differential form, with high-density (54 pairs) n-type/p-type single-crystal silicon thermocouples integrated within each thermopile (sample area ~0.045 mm^2^). The fabricated MEMS thermopile sensors exhibit outstanding power responsivity of 99.5 V/W and temperature responsivity of 27.8 mV/°C, which are more than 4 times higher than those reported for material thermal analysis. The high-responsivity MEMS DTA chips allow us to accurately measure the indium melting point at different heating rates of ~1–100 °C/s. We also perform DTA measurement of the dehydration process of CuSO_4_·5H_2_O and the crystals show three stages of losing water of crystallization before becoming anhydrous copper sulfate salt. Our high-performance, cost-effective MEMS sensing chips hold promise for rapid and accurate DTA characterization for a wide range of applications.

## 1. Introduction

Differential thermal analysis (DTA) is a prevailing analytical technique used in physics, chemistry, metallurgy, pharmaceuticals, food industries, and, more recently, nanoscience [1,2,3,4,5]. Through programmed heating and cooling, an analyte undergoes a chemical (e.g., oxidation, reduction, dehydration) or physical change (e.g., glass transition, melting) at a specific temperature with accompanying heat absorption or release, resulting in a temperature difference between the sample and the reference thermocouples [6,7]. Thus, the differential output signal from the thermocouples links the temperature with the specific thermodynamic properties of the substances and brings a better understanding of the physical and chemical processes of interest.

Traditional DTA apparatus consists of a pair of macroscopic thermocouples and heaters surrounding a crucible. The bulky systems suffer from low responsivity (~1–10 mV/W) and a slow heating rate (~2 °C/s) and consume mg-level samples, which hinder the DTA performance and efficiency [8,9]. Thanks to MEMS fabrication technology, the DTA core components can be significantly miniaturized into a chip-scale form, with heaters and thermocouples integrated into a single MEMS thermopile. These devices are typically fabricated on (100) or SOI wafers via double-sided fabrication to release the thermopiles from the backside. This cavity design allows good thermal insulation from the substrate to further improve temperature responsivity.

To date, MEMS thermopile sensors for the thermal analysis of materials have shown fast heating rates (e.g., ~1–1000 °C/s) and μg-level sample consumption [10]. However, as metal and polycrystalline silicon thermocouples are typically used, the current devices still show limited temperature responsivity of up to only ~3–6 mV/°C [11,12,13,14,15]. To continuously push for high-performance DTA, the temperature responsivity of MEMS thermopiles needs to be further improved. According to the Seebeck effect [16,17], a rational design for ultra-high responsivity MEMS thermopiles would be to integrate high-density, single-crystal silicon thermocouples into single thermocouples. It is well known that the Seebeck coefficient of single-crystal silicon can be much higher than that of polysilicon [18,19]. On the other hand, increasing the number of single-crystal silicon thermocouples will achieve more accumulated thermoelectric voltages when arranged in series.

In this work, we develop differential thermopile-based MEMS sensing chips for DTA. Facilitated by a unique “microholes interetch and sealing (MIS)” technique, the MEMS sensing chips with two identical and heating-programmable thermopiles are fabricated, and each thermopile consists of 54 pairs of thermocouples. More importantly, p-type and n-type single-crystal silicon are employed instead of polysilicon for the thermocouples. Due to the high Seebeck coefficient of single-crystal silicon and the high-density spiral design, our MEMS DTA chips exhibit outstanding power responsivity and temperature responsivity, which surpass those of current chips [11,12,13,14]. We also perform DTA measurements of the indium melting point and characterize the dehydration process of CuSO_4_·5H_2_O to validate our high-responsivity chip design.

## 2. Design of the MEMS Thermopile Sensors for DTA

**Figure 1:**
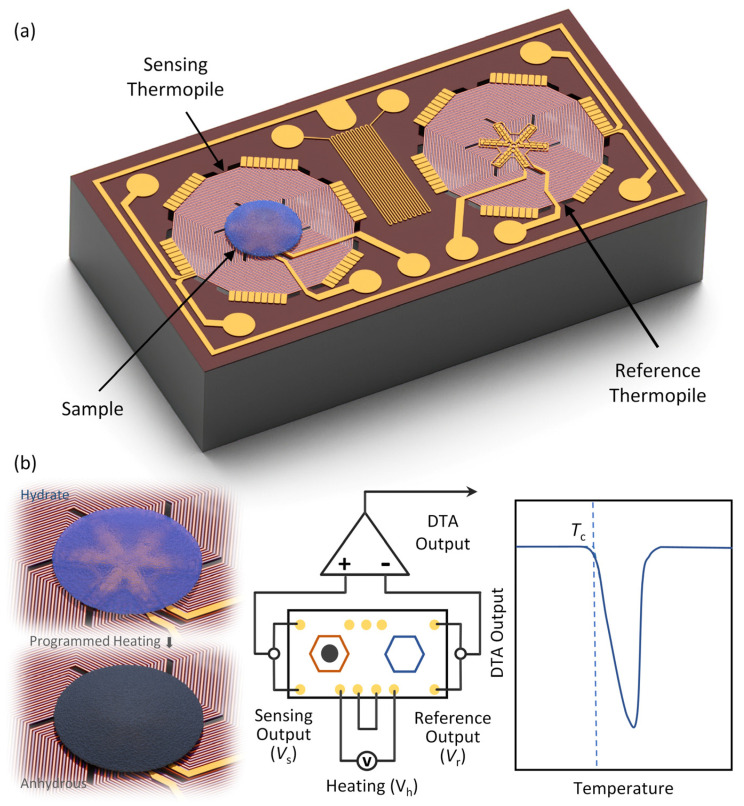
Design and working principle of the proposed MEMS thermopile-based DTA chip. (**a**) Schematic illustration of the MEMS DTA chip consisting of a pair of differential thermopiles for sensing and reference, respectively. (**b**) Schematic illustration of the working principle of the MEMS DTA chip. The sample is loaded within the sample area and heated. The temperature change caused by the endothermic (or exothermic) process is recorded as the DTA output.

The DTA chip (2 mm × 1 mm × 0.4 mm) is composed of a pair of identical MEMS thermopiles (i.e., sensing and reference thermopiles) in a differential form, as illustrated in Figure 1. The left thermopile (sensing thermopile) is loaded with the sample and detects the temperature change due to an endothermic (or exothermic) process, whereas the right thermopile (reference thermopile) is used as a reference. The differential measurement can eliminate the influence of common mode noise and environmental factors (e.g., heating voltage, ambient temperature, and flow rate of the air). Both thermopiles are heated by a programmed heating voltage.

Each thermopile is suspended on a silicon nitride (SiN_x_) adiabatic membrane over a thermal insulation cavity to avoid heat dissipation through the substrate so that the heat is only confined to the vicinity of the heating resistors. We design a total of 54 pairs of n-/p-type single-crystal silicon thermocouples in series within a ~640 µm diameter SiN_x_ membrane. In the center of the thermopile, a 240 µm diameter area is designed for the sample loading and DTA measurements. Each thermopile is also designed with a heating resistor around the hot junctions to heat the sample area uniformly. The hot junctions of the thermocouples are surrounded by the heating resistor in the sample area and the cold junctions are connected in parallel to the silicon frames (as the heat sink), which share the same temperature as the ambient. A single thermocouple can detect the temperature difference between the hot junction and the cold junction so that a thermopile can collect the average temperature above ambient within the sample area. Between two thermopiles, a Pt thermistor with a serpentine alignment is designed to detect the ambient temperature. We also arrange a metal guard ring around the entire device for noise shielding.

Figure 1b shows the working principle of the MEMS thermopile-based DTA chips. The analyte is loaded onto the sample area of the sensing thermopile and both thermopiles undergo a programmed heating process (by *V*_h_); the differential output voltage (i.e., DTA output) is recorded for analyzing the characteristic temperature of the analyte (e.g., melting, or dehydration temperature). According to the Seebeck effect, the relationship between the temperature difference and output voltage can be expressed as

(1) $$V=N\left(\alpha_{A}-\alpha_{B}\right)\Delta T$$

where *N* is the number of thermocouple pairs, *α_A_*_,_ and *α_B_* present the Seebeck coefficients of the p- and n-type single-crystal silicon that form hot junctions, and Δ*T* is the temperature difference between the sample area and the environment. For the DTA chip, the output voltage can be expressed as

(2) $$DTA\;Output=V_{s}-V_{r}=N\left(\alpha_{A}-\alpha_{B}\right)\Delta T_{sample}$$

where Δ*T*_sample_ is the temperature change caused by the endothermic (or exothermic) process.

To improve the DTA performance while reducing the chip size, we enhance the power and temperature responsivity of the thermopile by designing high-density single-crystal silicon thermocouples with a significant Seebeck coefficient. Meanwhile, thanks to the high thermal conductivity of the single-crystal silicon (~140 W/m·K), the chip achieves a shorter time constant. Together with the thermal insulation cavity, the chips achieve a much higher resolution for detecting temperature variations of ~0.05 mK, which is described in Section 4.

## 3. Fabrication of the MEMS Thermopile Sensors for DTA

**Figure 2:**
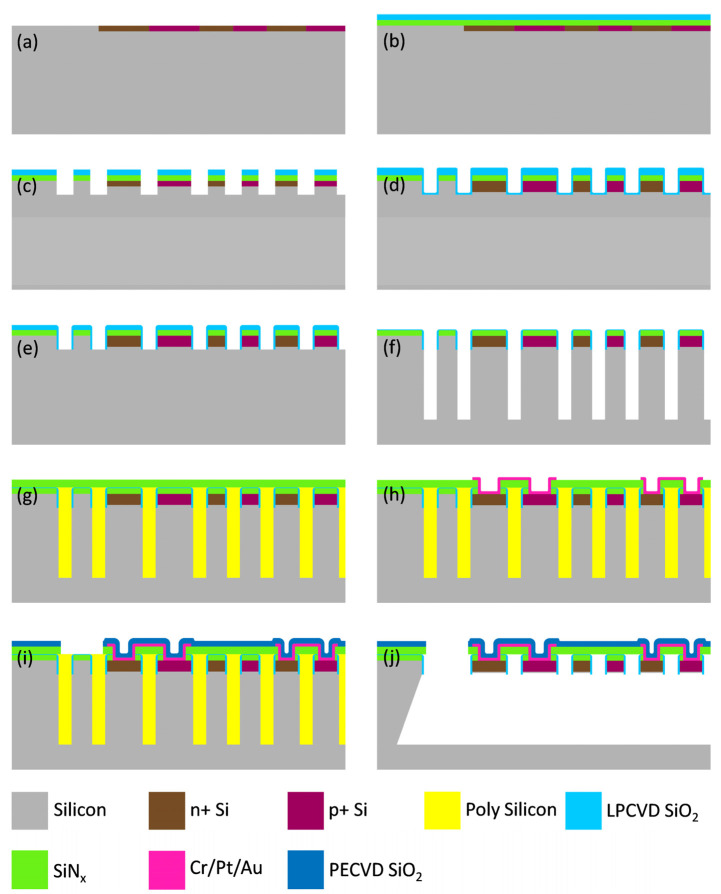
The fabrication process of the MEMS thermopile sensors for DTA. (**a**) N-type and p-type ion implantation. (**b**) Dielectric film deposition. (**c**) Thermocouple patterning and thickness definition. (**d**) Sidewall protection layer deposition and diffusion. (**e**) Sidewall protection definition. (**f**) DRIE etching. (**g**) Trench-filling with polysilicon, self-stopping polishing, and supporting film deposition. (**h**) Contact hole patterning, metal sputtering, and electrode patterning. (**i**) SiO_2_ deposition and release hole patterning. (**j**) TMAH wet etching.

It is challenging to batch fabricate suspended single-crystal silicon thermopiles for DTA application. Polysilicon has often been used in the fabrication of MEMS thermocouples on (100) wafers and it is hard to control the silicon thickness by the deep and time-consuming backside wafer etching. On the other hand, although SOI wafers can directly provide a single-crystal silicon layer to form thermocouples, the thickness of the silicon layer is highly non-uniform. Based on a “microholes interetch and sealing” (MIS) technique from our group [20,21], we develop a fabrication process of single-crystal silicon thermopiles using (111) wafers. Taking advantage of the anisotropic wet-etching properties of single-crystal silicon, the MIS process provides uniform silicon etching after the sidewall protection and microhole sealing steps. High-density, spirally arranged single-crystal silicon beams are easily suspended over a thermal insulation cavity only from the front side of the wafer. The MIS technology uses non-SOI wafers and a CMOS-compatible single-sided process to fabricate MEMS devices with complex single-crystal silicon structures, offering advantages in uniformity (e.g., device thickness) and reducing manufacturing costs. The fabrication steps of the MEMS thermopile-based DTA chips start with a single-side-polished 4-inch (111) wafer. The detailed steps are illustrated in Figure 2 and are as follows:

1. First, phosphorous ions and boron ions are area-selectively implanted to create n-type and p-type single-crystal silicon thermocouples, as shown in Figure 2a. The implantation energy of the phosphorus and boron ions is kept at ~50 keV, with doses of 8 × 10^15^ and 1 × 10^16^ ion/cm^2^, respectively.
2. A 0.5 μm thick low-stress silicon nitride (SiN_x_) film and 0.4 μm thick tetraethyl orthosilicate (TEOS) silicon oxide (SiO_2_) film are deposited by low-pressure chemical vapor deposition (LPCVD), as shown in Figure 2b.
3. The thermocouples are patterned by photolithography and the SiN_x_ and SiO_2_ are patterned to expose the bulk silicon by reactive ion etching (RIE). Subsequently, the bulk silicon is etched to 4 μm to determine the thickness of the single-crystal silicon thermocouple with deep reactive ion etching (DRIE), as shown in Figure 2c.
4. SiO_2_ film with a 0.4 μm thickness is deposited by LPCVD. Compared to SiN_x_, SiO_2_ can protect the sidewall against the DRIE etching, especially avoiding excessive etching of the corner of the dielectric layer at the top of the sidewall (Figure 2d).
5. SiO_2_ with a thickness of 0.4 um is anisotropically etched by RIE to form the sidewall structure, which is used to protect the single-crystal silicon thermocouple beams against the wet etching in the final step, as illustrated in Figure 2e.
6. Through a maskless, self-aligned process, deep trenches of 40 μm are etched using DRIE to define the depth of the thermally isolated cavity (Figure 2f). During the deep silicon etching process, the SiO_2_ on the surface is consumed uniformly without causing depressions.
7. Polysilicon with a thickness of 2.5 μm is deposited using LPCVD to fill the deep trenches and then polished to expose the SiO_2_ surface using chemical mechanical polishing (CMP). By using a polishing solution with a removal ratio of over 100:1 for polysilicon vs. SiO_2_, the CMP stops once the surface of SiO_2_ is exposed to achieve a self-stopping flattening. Then, SiN_x_ with a thickness of 1μm is deposited by LPCVD as the supporting membrane for the single-crystal silicon thermocouples.
8. The contact holes of the single-crystal silicon thermocouples are patterned by removing the SiN_x_ using RIE. The interconnects between the thermocouples are performed by sputtering and patterning Cr/Pt/Au metal films with thicknesses of 40 nm/100 nm/3000 nm, respectively (Figure 2h).

9. SiO_2_ with a thickness of 0.5 μm is deposited using plasma-enhanced chemical vapor deposition (PECVD) as the metal insulating layer. Then, the release holes are patterned using RIE until the polysilicon is exposed (Figure 2i).
10. The 25% tetramethylammonium hydroxide (TMAH) at 80 °C is used to perform the anisotropic wet etching of the (111) silicon wafers, forming a thermal insulation cavity and suspending the thermopiles.

Since etching polysilicon by TMAH is isotropic and the etch rate is faster, the etching process first proceeds vertically along the deep etches filled with polysilicon. Then the etchant horizontally removes the single-crystal silicon on both sides of the deep trenches. Compared with traditional frontside and backside etching, the release time of the entire device is shortened from several hours to ~30 min, which makes the release times across the device roughly the same and avoids excessive etching of the thermocouple beams near the release holes.

## 4. Characterization of the MEMS Thermopile Sensors

**Figure 3:**
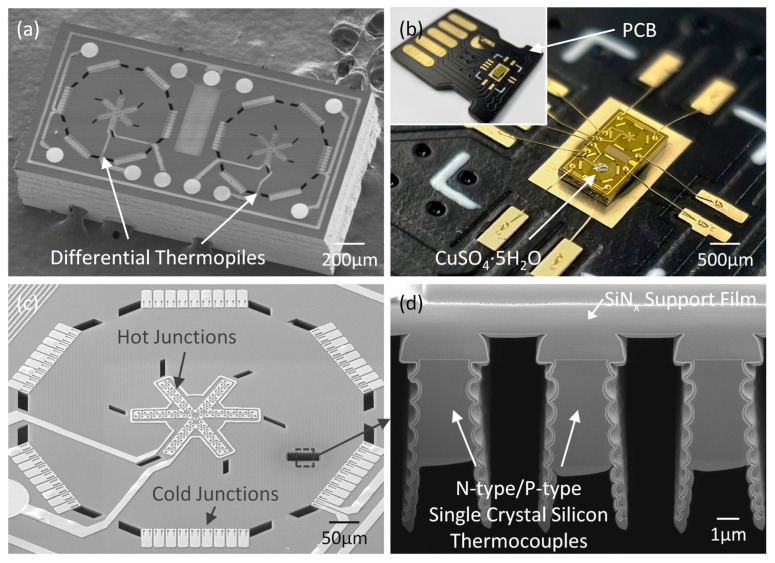
Image of the fabricated MEMS thermopile chips. (**a**) SEM image showing a pair of differential thermopiles on a 2 mm × 1 mm × 0.4 mm die. (**b**) Optical image of the MEMS thermopile chip after wire bonding and sample loading. (**c**) Zoomed-in view of the single thermopile showing 54 pairs of single-crystal thermocouples arranged in a spiral shape, with hot junctions in the center and cold junctions at the edges. (**d**) Cross-sectional view of the single-crystal silicon thermocouples underneath the SiN_x_ film.

The fabricated MEMS thermopiles are characterized using scanning electron microscopy (SEM), as illustrated in Figure 3a. The chip is 2 mm × 1 mm × 0.4 mm and consists of a pair of single-crystal silicon thermopiles, i.e., a sensing thermopile on the left and a reference thermopile on the right. Figure 3b shows a typical device after wire bonding and sample loading with CuSO_4_·5H_2_O. Figure 3c shows the details of a single thermopile. We achieve a total of 54 pairs of n-type/p-type single-crystal silicon thermocouples in a spiral shape within a ~640 µm diameter area (~0.32 mm^2^) on each thermopile. The hot junctions are surrounded by a heating resistor and are distributed in the device center within a ~240 µm diameter area (~0.045 mm^2^), i.e., the sample area. The cold junctions are arranged at the edges. The surface of each thermopile is relatively flat after release. Focus Ion Beam (FIB) is used to cut the SiN_x_ film and provide a cross-sectional view of the thermocouples. As shown in Figure 3d, n-type/p-type single-crystal silicon thermocouples with a width of 3 μm are observed attached under the 1μm thick SiN_x_ supporting film.

## 5. Thermal Response of the MEMS Thermopile Sensors

**Figure 4:**
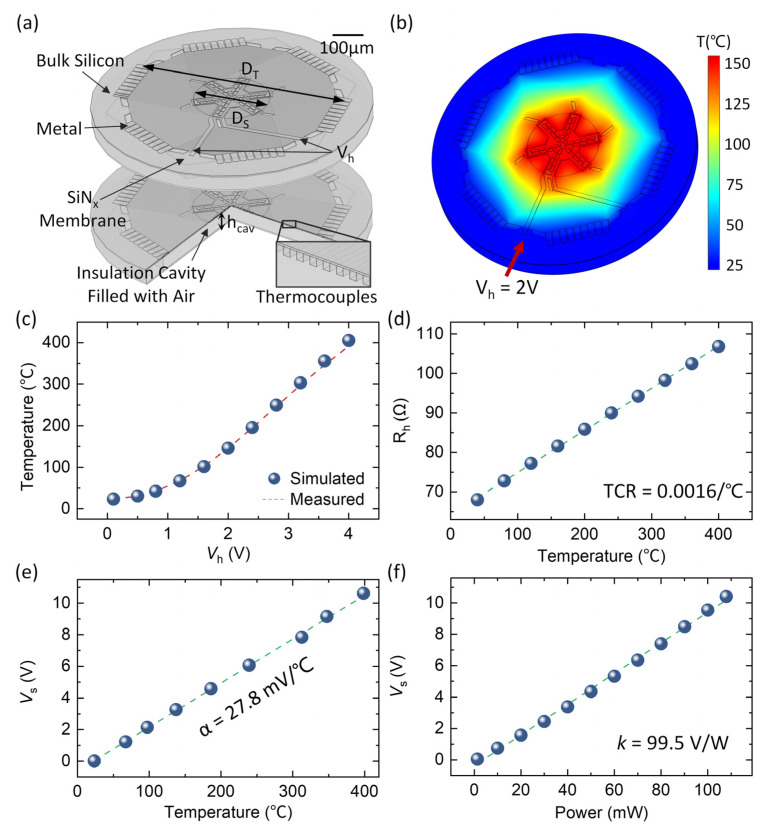
Thermal response of the MEMS thermopile chips. (**a**) The 3D model for finite-element simulation. (**b**) Temperature distribution at a heating voltage (*V*_h_) of 2V. (**c**) Heating voltage (*V*_h_) vs. the average temperature in the sample area. The measured results agree with the finite-element simulation results. (**d**) Measured heating resistance (R_h_) vs. average temperature in the sample area, which indicates a TCR of 0.0016/°C. (**e**) The output voltage of the sensing thermopile (*V*_s_) vs. the average temperature in the sample area, which shows an ultra-high temperature responsivity of 27.8 mV/°C. (**f**) The output voltage of the sensing thermopile (*V*_s_) vs. the heating power, which shows an ultra-high power responsivity of 99.5 V/W.

We first calibrate the thermal response (including temperature response and power response) of an individual MEMS thermopile (we measure a sensing thermopile in Figure 4). The thermopile is heated by applying voltage (*V*_h_) to the heating resistor (*R*_h_) and the heating current (*I*_h_) is recorded. The heating voltage (*V*_h_) is set from 0 V to 4 V and the average temperature within the sample area is measured non-contact by the infrared camera. The heating power applied to the thermopile is calculated by *P* = *V*_h_*I*_h_.

We also perform a finite-element simulation to model the temperature distribution of the thermopile. A 3D thermopile model is established, including 54 pairs of single-crystal silicon thermocouples arranged in a shape consistent with the design, a 1μm thick SiN_x_ suspension support film, and a metal layer for contact at the center and edges of the thermopile, as shown in Figure 4a. A single-crystal silicon substrate and a 40μm thick air cavity between the thermocouple and the substrate for thermal insulation are also considered in the model such that both heat transfer through the solid and convection by the air are involved. A forced convective heat transfer on the upper surface of the device is set. To reduce the simulation time without compromising accuracy, a fixed convective heat dissipation coefficient is used instead, and the value we use is close to the reported value in the literature [22]. The structural dimensions of the device and the thermophysical parameters of the materials are listed in Table 1. With Joule heating at the device center, the relationship between the heating voltage and the average temperature within the sample area is predicted, as shown in Figure 4b. According to the simulation results, the temperature variation within the sample area is < ±3% up to 400 °C. Therefore, our design ensures a uniform temperature distribution in the sample area for the DTA measurements.

Figure 4c shows the measured average temperature in the sample area as a function of the heating voltage (*V*_h_). The experimental results agree with the simulation results, which verifies our sensor design. We then measure the relationship between the heating resistance and average temperature in the sample area, as shown in Figure 4d, which indicates a TCR of ~0.0016/°C for the heating resistor.

To calibrate the temperature and power responsivity of the MEMS thermopiles, the output voltage of the sensing thermopile (*V*_s_) and the heating power are recorded simultaneously. As shown in Figure 4e, the thermopile exhibits a linear response to the temperature in the sample area with an ultra-high temperature responsivity of *S*_T_ = 27.8 mV/°C. A single thermopile can output up to 6 V at a temperature difference of 215°C (absolute temperature of 240 °C at room temperature). The MEMS thermopiles also show an ultra-high power responsivity of *S*_P_ = 99.5 V/W, as shown in Figure 4f.

Besides calibrating the thermal responses of the fabricated MEMS thermopiles, we also estimate the thermal time constant (τ), noise equivalent temperature (NET), and noise equivalent power (NEP). The thermal time constant (τ) of the MEMS thermopiles is given by the following equation [23]:

(3) $$\tau =\frac{1}{4f_{c}}$$

Given a measured −3 dB cut-off frequency (*f*_c_) of ~108 Hz for our devices, the time constant can be estimated as ~2.3 ms. The root-mean-square (rms) voltage noise of the MEMS thermopiles can be expressed as [23]:

(4) $$V_{noise,rms}=\sqrt{4k_{B}TRB}$$

where *k*_B_ is the Boltzmann’s constant (1.38 × 10^−23^ J/K), *T* is the room temperature (300 K), *R* is the device output resistance (540 kΩ), and *B* is the system bandwidth of 400 Hz [11]. Therefore, we calculate the *V*_noise,rms_ = 1.9 µV for our MEMS thermopiles. Given the temperature responsivity of *S*_T_ = 27.8 mV/K and the power responsivity of *S*_P_ = 99.5 V/W, the NET and NEP can be estimated as 0.068 mK and 0.019 μW, respectively. Considering the calculation method in Ref. [11], where the NET and NEP are calculated based on 8×*V*_noise, rms_, an NET of ~0.54 mK and NEP of ~0.15 μW can also be estimated.

Table 2 compares the MEMS thermopile chips for thermal analysis of materials in this work and the literature. Compared to the reported devices, our MEMS thermopiles chips for thermal analysis of materials exhibit more than four times higher temperature responsivity and power responsivity, benefitting from the single-crystal silicon thermocouples and their high-density arrangement. Meanwhile, our devices have comparable low noise and the shortest thermal time constant. The outstanding performance of our MEMS thermopile sensors ensures accurate and rapid DTA measurements, even with a small amount of sample < 1 µg, which is detailed in the next section.

## 6. DTA Measurements Using MEMS Thermopile Sensors

In this section, our MEMS thermopile sensors are tested by performing DTA measurements of the indium melting and the dehydration of the copper sulfate pentahydrate.

To achieve high-performance DTA measurements, we design an analog front-end circuit. The dynamic range of the MEMS thermopiles is up to 85 dB (from NET to 400 °C), which requires a large dynamic range, a high-precision signal readout, and a processing circuit. A 24-bit ADC (AD7134, Analog Devices, Inc., Wilmington, MA, USA) is used to acquire the data, providing a dynamic range of ~120 dB. The DTA output is amplified ten times to reduce the circuit noise, whereas the output voltage of both thermopiles (*V*_s_ and *V*_r_) is reduced by a factor of 3.75 to fit the measurement range of the ADC. A second-order Butterworth filter is used to limit the bandwidth of the signal to 400 Hz to reduce noise. The heating voltage (*V*_h_) is generated by a 16-bit DAC (LT2664, Analog Devices, Inc., Wilmington, MA, USA) with a resolution of 300 μV and a voltage settling time of ~10 μs. A switching power supply and low dropout linear regulators (LDO) are used to provide low-noise power rails instead of commercial DC voltage supplies. The ADC and DAC are controlled by FPGA, which provides automated control including data transfer, voltage control, and programmed heating. Finally, the FPGA is connected to a PC via a local area network (LAN) and the PC software saves the DTA curves.

The MEMS thermopile sensor is mounted on the sample stage under an optical microscope so that the morphological changes can be imaged during the DTA measurements. We utilize a microinjection system to load the samples precisely onto the sensing area of the sensing thermopile.

### 6.1. DTA Measurement of Indium Melting

**Figure 5:**
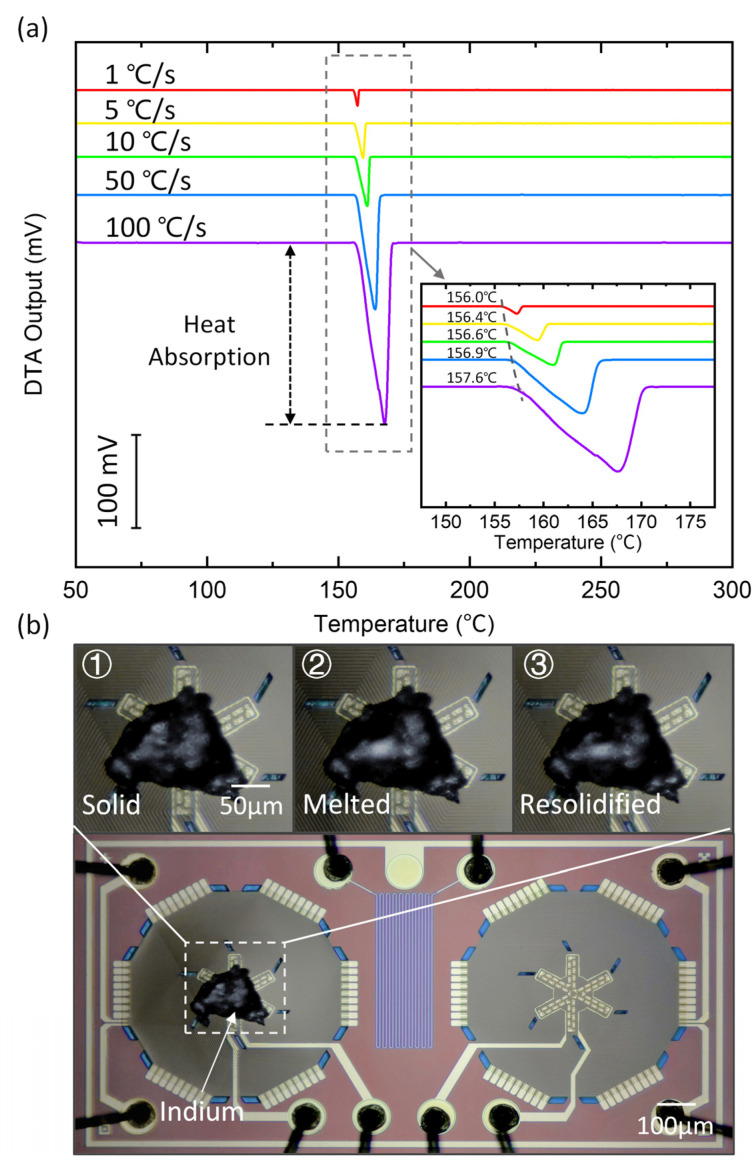
DTA measurement of indium melting using the MEMS thermopile sensors. (**a**) DTA output taken at different heating rates of 1–100 °C/s; the measured melting point is consistent with the reported value (156.6 °C), with a small thermal lag within 1 °C up to 100 °C/s. (**b**) Optical images showing the melting and resolidification of indium.

We first perform a DTA measurement of the indium melting process to validate that our MEMS thermopile sensors can be used to characterize the melting point of a solid. A piece of indium (Thermal Analysis-Indium, Aladdin, Shanghai, China) with an area of ~0.015 mm^2^ (sample mass of ~0.5 µg) is cut under a microscope and loaded onto the sample area of the sensing thermopile by the capillary of the microinjection system, as shown in Figure 5b. The MEMS thermopiles are heated at different heating rates (1 °C/s–100 °C/s) while the DTA output signals are recorded.

The DTA outputs at different heating rates are shown in Figure 5a. The peak toward the negative axis direction suggests that indium melting is a heat absorption process. The peak value is ~190 mV (corresponding to a temperature change of 6.8 °C), which validates the ultra-high responsivity of the MEMS thermopile sensors. The measured melting point is 156.0 °C at a heating rate of 1 °C/s and 157.6 °C at a heating rate of 100 °C/s, which shows a thermal lag of within 1 °C compared to the reported value of 156.6 °C [24]. Figure 5b shows detailed images of the indium melting and resolidification processes during the DTA measurement. We can see that the indium is solid at room temperature and suddenly becomes liquified when reaching the melting point. The indium then solidifies again after the DTA measurement.

### 6.2. DTA Measurement of the Dehydration Process of Copper Sulfate Pentahydrate

Copper sulfate pentahydrate (CuSO_4_·5H_2_O) is a hydrated mineral salt widely used in paints, batteries, and other chemical applications [25]. CuSO_4_·5H_2_O crystals are blue and turn into an opaque white powder after losing water of crystallization. Therefore, the characterization of the multi-step dehydration process of CuSO_4_·5H_2_O [26,27] is a good choice to validate the high responsivity and accuracy of our MEMS thermopile sensors for DTA. CuSO_4_·5H_2_O is prepared in a saturated aqueous solution and the saturated solution is loaded by the capillary of the microinjection system to cover the sample area fully. After the water gradually evaporates at room temperature, CuSO_4_·5H_2_O is crystalized under an optical microscope. As a single droplet of saturated solution ~3.6 nL is loaded on the sensing thermopile, the mass of the loaded CuSO_4_·5H_2_O is estimated to be ~0.8 μg.

**Figure 6:**
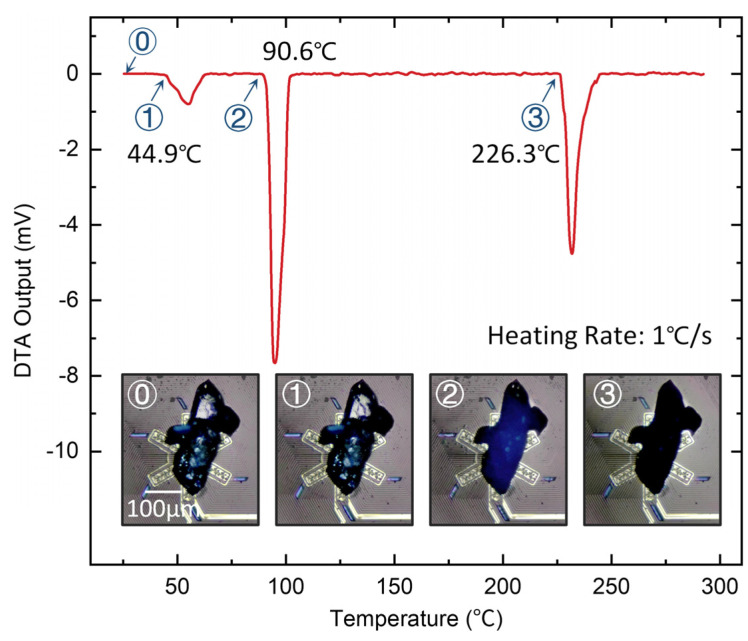
DTA measurement of the dehydration process of CuSO_4_·5H_2_O using the MEMS thermopile sensors. The dehydration process can be divided into three stages at temperatures of 44.9°C, 90.6°C, and 226.3°C. The optical images show transparency change when CuSO_4_·5H_2_O gradually becomes CuSO_4_.

The DTA output at a heating rate of 1 °C/s is shown in Figure 6. We measure three heat absorption processes at temperatures of 44.9 °C, 90.6 °C, and 226.3 °C, respectively, which correspond to the three stages of water of crystallization loss before the crystals eventually become anhydrous copper sulfate salt (CuSO_4_). The optical images also show the color change before and after CuSO_4_·5H_2_O dehydration. Our experimental results agree with the reported results measured at a heating rate of 0.016 °C/s using conventional DTA instruments [25]. At a faster heating rate (~0.5 °C/s), the traditional DTA instrument cannot resolve two heat absorption peaks within 40–100 °C. Thus, for the DTA measurement of the three-step CuSO_4_·5H_2_O dehydration at 0.016 °C/s, heating from room temperature to 400 °C would take ~6.5 h for a ~5 mg sample using a conventional DTA instrument. In contrast, it only takes ~6.5 min to characterize the three-step CuSO_4_·5H_2_O dehydration using a < 1 µg sample.

Overall, our measurement results confirm that our MEMS thermopile sensors can be used for accurate and rapid DTA of materials.

## 7. Conclusions

In summary, we have designed and fabricated MEMS thermopile sensors for differential thermal analysis. Differential thermopiles are batch fabricated with high-density n-type/p-type single-crystal silicon thermocouples using the “microholes interetch and sealing (MIS)” technique. Due to the high Seebeck coefficient of single-crystal silicon and the high-density spiral design, the sensors have exhibited outstanding temperature responsivity of 27.8 mV/°C and power responsivity of 99.5 V/W, which is more than a four-fold improvement compared with other MEMS thermopile-based DTA chips. We have also performed DTA measurements of indium melting at different heating rates of 1–100 °C/s, showing good accuracy in determining the indium melting point. Meanwhile, in the DTA measurement of the dehydration process of CuSO_4_·5H_2_O, we have observed three stages of water of crystallization loss before the crystals eventually became anhydrous copper sulfate salt. Our MEMS DTA chips are expected to be operated at heating rates in the range of ~1 °C/s–1000 °C/s. A lower heating rate results in reduced heat absorption and increased heat dissipation during the measurements, leading to a very weak output signal. On the other hand, heating rates above 1000 °C/s may drastically increase the thermal lag, resulting in inaccurate DTA measurements. In addition, due to the miniature device dimensions and high sensitivity in measuring the temperature changes, the sample is expected to be at the μg level and covers the sensing area. Large amounts of the sample may not be heated evenly on the DTA chip, especially under fast heating rates, causing thermal lag and errors in determining the characteristic temperatures. Our ultra-high responsivity MEMS thermopile sensors hold promise for rapid and accurate DTA in a wide range of applications in physics, chemistry, metallurgy, pharmaceuticals, and nanoscience. Besides the dehydration and metal melting that have been demonstrated in this work, we expect that our MEMS DTA chips can also be used for temperature characterizations of other chemical processes (e.g., oxidation, reduction) or physical changes (e.g., glass transition). Moreover, the fast heating rate greatly reduces the thermal analysis time and improves test efficiency.

## Figures and Tables

**Table 1:** Model structural dimensions and material thermophysical parameters.

Parameters	Value
Diameter of thermopile (D_T_) (μm)	640
Diameter of sensing area (D_S_) (μm)	240
Thickness of thermocouples (μm)	4
Width of thermocouples (μm)	3
Thickness of SiN_x_ membrane (μm)	1
Thickness of metal (μm)	0.4
Height of isolation cavity (h_cav_) (μm)	40
Thermal conductivity of silicon (W/(m·K))	130
Thermal conductivity of SiN_x_ (W/(m·K))	4
Thermal conductivity of metal (W/(m·K))	36
TCR of metal (1/K)	0.0016
Convection heat transfer coefficient (W/(m^2^·K))	220
Boundary temperature (K)	293
Initial temperature (K)	293

**Table 2:** Comparison of MEMS differential thermopiles for thermal analysis of materials.

Reference	This Work	[11]	[12]	[13]	[14]
Size (mm^2^)	2 × 1	5 × 3.3	/	5 × 3	20 × 15
Thermocouple Material	single-crystal Si	polysilicon	Cr-Ni	polysilicon	Sb-Bi
# of Thermocouple pairs	54	8	6	8	50
Sample Area (mm^2^)	0.045	0.2	0.033	0.2	5
*V*_noise,rms_ (µV)	1.9	0.3	5	0.26	0.2
*S*_T_ (mV/K)	27.8	4	0.03	2.9	6.3
*S*_P_ (V/W)	99.5	24	0.15	23	4
NET (mK) ^a^	0.068 ^b^	0.075 ^b^	170	0.09	0.03
NEP (µW) ^a^	0.019 ^b^	0.013 ^b^	33	0.011 ^b^	0.05
τ (ms)	2.3	12	5.3	/	2800

^a^ These values are calculated using *V*_noise,rms._^b^ These values are calculated with a bandwidth of 400 Hz for comparison.

## Data Availability

Not applicable.

## References

[1] Day R.P., Xia J., Petibon R., Rucska J., Wang H., Wright A.T.B., Dahn J.R. (2015). Differential Thermal Analysis of Li-Ion Cells as an Effective Probe of Liquid Electrolyte Evolution during Aging. J. Electrochem. Soc..

[2] Lavor E.P., Navarro M.V.M., Freire F.D., Aragão C.F.S., Raffin F.N., Barbosa E.G., de Lima e Moura T.F.A. (2014). Application of Thermal Analysis to the Study of Antituberculosis Drugs–Excipient Compatibility. J. Therm. Anal. Calorim..

[3] Bernhard M., Presoly P., Bernhard C., Hahn S., Ilie S. (2021). An Assessment of Analytical Liquidus Equations for Fe-C-Si-Mn-Al-P-Alloyed Steels Using DSC/DTA Techniques. Metall. Mater. Trans. B.

[4] Samaržija-Jovanović S., Jovanović V., Konstantinović S., Marković G., Marinović-Cincović M. (2010). Thermal Behavior of Modified Urea–Formaldehyde Resins. J. Therm. Anal. Calorim..

[5] Abd-Elnaiem A.M., Abbady G. (2020). A Thermal Analysis Study of Melt-Quenched Zn_5_Se_95_ Chalcogenide Glass. J. Alloy. Compd..

[6] Haines P.J., Reading M., Wilburn F.W., Brown M.E. (1998). Chapter 5—Differential Thermal Analysis and Differential Scanning Calorimetry. Handbook of Thermal Analysis and Calorimetry.

[7] Gong X., Guo Z., Wang Z. (2010). Variation on Anthracite Combustion Efficiency with CeO_2_ and Fe_2_O_3_ Addition by Differential Thermal Analysis (DTA). Energy.

[8] Schubert F., Gollner M., Kita J., Linseis F., Moos R. (2016). First Steps to Develop a Sensor for a Tian–Calvet Calorimeter with Increased Sensitivity. J. Sens. Sens. Syst..

[9] Bin T., Qu J., Liu L., Feng Y., Hu S., Yin X. (2011). Non-Isothermal Crystallization Kinetics and Dynamic Mechanical Thermal Properties of Poly(Butylene Succinate) Composites Reinforced with Cotton Stalk Bast Fibers. Thermochim. Acta.

[10] van Herwaarden A.W. (2005). Overview of Calorimeter Chips for Various Applications. Thermochim. Acta.

[11] van Herwaarden S., Iervolino E., van Herwaarden F., Wijffels T., Leenaers A., Mathot V. (2011). Design, Performance and Analysis of Thermal Lag of the UFS1 Twin-Calorimeter Chip for Fast Scanning Calorimetry Using the Mettler-Toledo Flash DSC 1. Thermochim. Acta.

[12] Nakabeppu O., Deno K. (2016). Nano-DTA and Nano-DSC with Cantilever-Type Calorimeter. Thermochim. Acta.

[13] Zhou W., Li X., Yao F., Zhang H., Sun K., Chen F., Xu P., Li X. (2022). Chip-Based MEMS Platform for Thermogravimetric/Differential Thermal Analysis (TG/DTA) Joint Characterization of Materials. Micromachines.

[14] Wang B., Lin Q. A MEMS Differential Scanning Calorimeter for Thermodynamic Characterization of Biomolecules. Proceedings of the 2011 IEEE 24th International Conference on Micro Electro Mechanical Systems.

[15] Zhuravlev E., Schick C. (2010). Fast Scanning Power Compensated Differential Scanning Nano-Calorimeter: 1. The Device. Thermochim. Acta.

[16] Geballe T.H., Hull G.W. (1955). Seebeck Effect in Silicon. Phys. Rev..

[17] Stranz A., Kähler J., Waag A., Peiner E. (2013). Thermoelectric Properties of High-Doped Silicon from Room Temperature to 900 K. J. Elec. Materi..

[18] Allison S.C., Smith R.L., Howard D.W., González C., Collins S.D. (2003). A Bulk Micromachined Silicon Thermopile with High Sensitivity. Sens. Actuators A Phys..

[19] Xie J., Lee C., Wang M.-F., Liu Y., Feng H. (2009). Characterization of Heavily Doped Polysilicon Films for CMOS-MEMS Thermoelectric Power Generators. J. Micromech. Microeng..

[20] Wang J., Li X. (2011). Single-Side Fabricated Pressure Sensors for IC-Foundry-Compatible, High-Yield, and Low-Cost Volume Production. IEEE Electron. Device Lett..

[21] Wang J., Xia X., Li X. (2012). Monolithic Integration of Pressure Plus Acceleration Composite TPMS Sensors With a Single-Sided Micromachining Technology. J. Microelectromech. Syst..

[22] Pranti A.S., Loof D., Kunz S., Zielasek V., Bäumer M., Lang W. (2019). Ligand-Linked Nanoparticles-Based Hydrogen Gas Sensor with Excellent Homogeneous Temperature Field and a Comparative Stability Evaluation of Different Ligand-Linked Catalysts. Sensors.

[23] Graf A., Arndt M., Sauer M., Gerlach G. (2007). Review of Micromachined Thermopiles for Infrared Detection. Meas. Sci. Technol..

[24] Failleau G., Fleurence N., Morice R., Gaviot E., Rénaot E. (2010). Adiabatic Calorimetry Approach to Assess Thermal Influences on the Indium Melting Point. Int. J. Thermophys..

[25] Cheng L., Li W., Li Y., Yang Y., Li Y., Cheng Y., Song D. (2019). Thermal Analysis and Decomposition Kinetics of the Dehydration of Copper Sulfate Pentahydrate. J. Therm. Anal. Calorim..

[26] Hevroni L., Shamish Z., Danon A. (2009). Thermal Dehydration and Decomposition of Copper Selenate Pentahydrate. J. Therm. Anal. Calorim..

[27] El-Houte S., El-Sayed Ali M., Sørensen O.T. (1989). Dehydration of CuSO_4_ · 5H_2_O Studied by Conventional and Advanced Thermal Analysis Techniques. Thermochim. Acta.

